# Supplementary material for: Quantitative proteomic analysis identified differentially expressed proteins with tail/rump fat deposition in Chinese thin- and fat-tailed lambs
Source: PLoS One. 2021 Feb 2;16(2):e0246279. doi: 10.1371/journal.pone.0246279 (PMC7853479; doi:10.1371/journal.pone.0246279)
Supplement: S1 Fig — (A) H&E staining for the TAT; (B) Cell diameter analysis. Cell diameter unit was μm, bars without a common letter differ, P < 0.05. (DOC) [file pone.0246279.s001.doc]

**
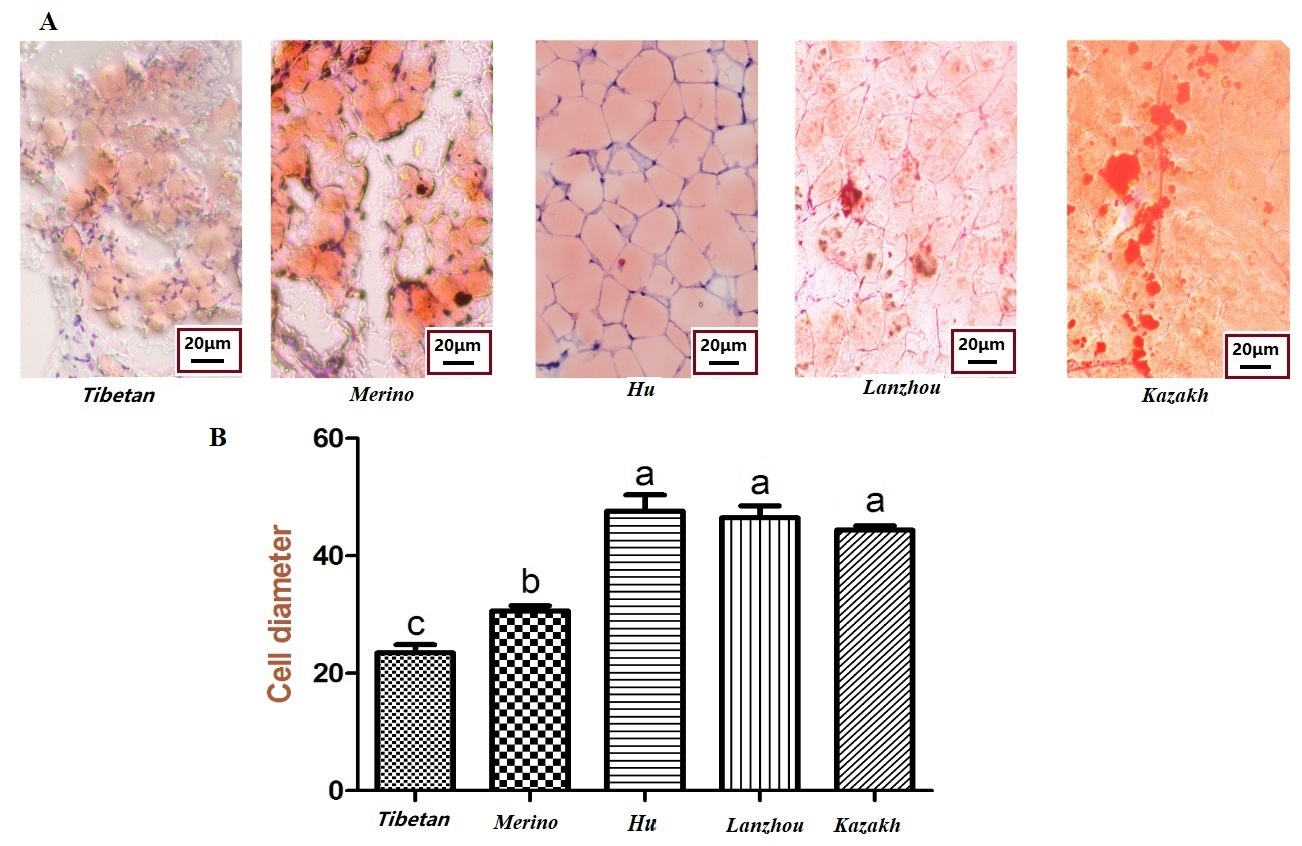
**

**S1 Fig.** Adipocyte diameter comparative analysis of TAT in the five breeds.

(A) H&E staining for the TAT; (B) Cell diameter analysis. Cell diameter unit was μm, bars without a common letter differ, *P* < 0.05.
