# Supplementary material for: Quantitative proteomic analysis identified differentially expressed proteins with tail/rump fat deposition in Chinese thin- and fat-tailed lambs
Source: PLoS One. 2021 Feb 2;16(2):e0246279. doi: 10.1371/journal.pone.0246279 (PMC7853479; doi:10.1371/journal.pone.0246279)
Supplement: S2 Fig — PA means protein areas. K represent Kazakh sheep, L represent Lanzhou big tailed sheep, H represent Hu sheep, M represent Alpine Merino sheep, T represent Tibetan sheep. (DOC) [file pone.0246279.s002.doc]

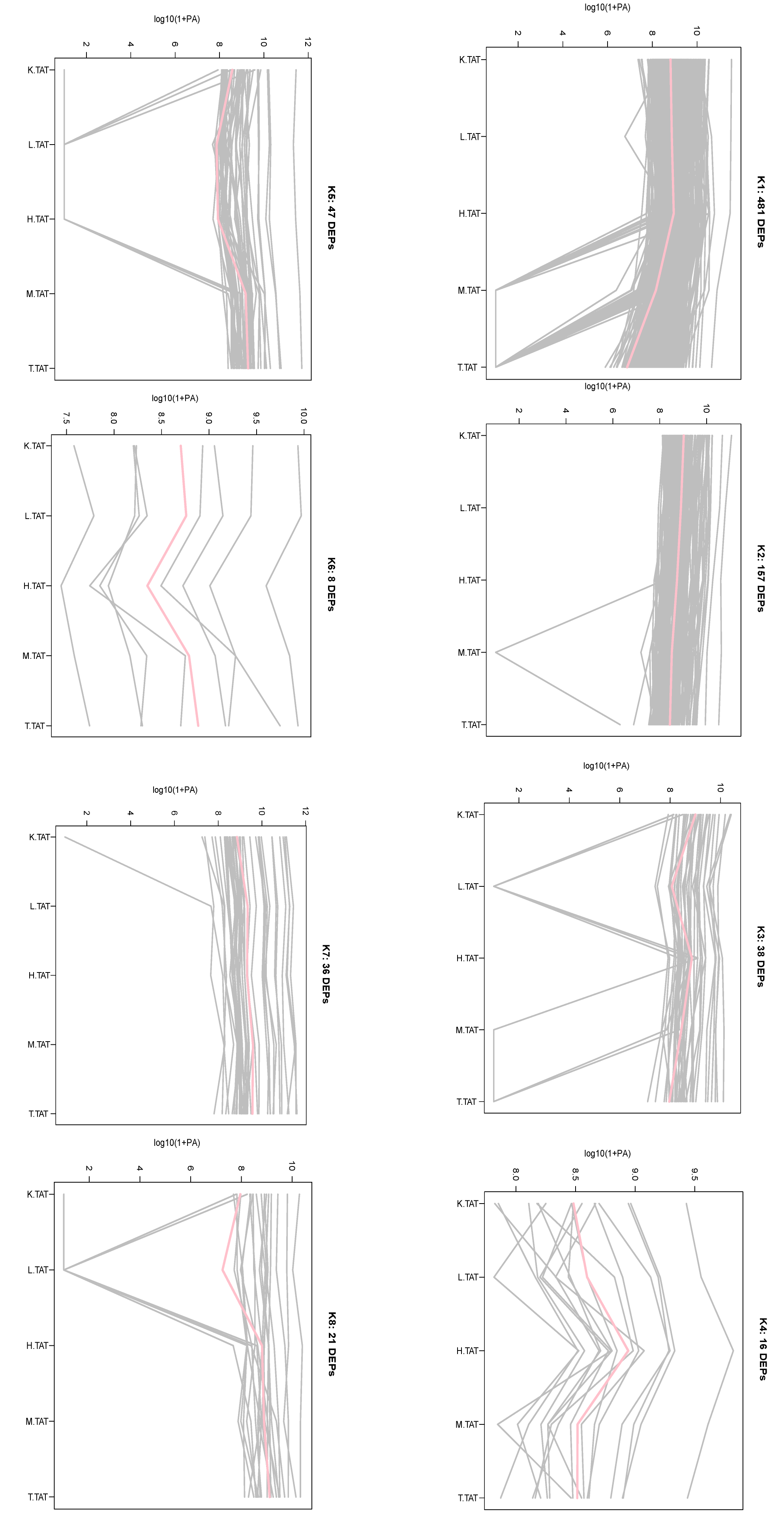


**S2 Fig.** The eight cluster of the DEPs.

PA means protein areas. K represent Kazakh sheep, L represent Lanzhou big tailed sheep, H represent Hu sheep, M represent Alpine Merino sheep, T represent Tibetan sheep.
