# Supplementary material for: Quantitative proteomic analysis identified differentially expressed proteins with tail/rump fat deposition in Chinese thin- and fat-tailed lambs
Source: PLoS One. 2021 Feb 2;16(2):e0246279. doi: 10.1371/journal.pone.0246279 (PMC7853479; doi:10.1371/journal.pone.0246279)
Supplement: S3 Fig — 18 proteins were selected, with GAPDH as the internal reference gene. The P values of relative expression of gene and its protein: 0.1526(AACS) and 0.0087 (AACS); 0.0192 (ACACA) and 0.0086 (ACACA); 0.0337 (ACADVL) and 0.0091 (ACADVL); 0.0171 (ACSS2) and 0.0087 (ACSS2); 0.0197 (ADIPOQ) and 0.0101 (ADIPOQ); 0.0477 (ADIRF) and 0.0117 (ADIRF); 0.0337 (ASPN) and 0.0101 (ASPN); 0.0302 (ELOVL6) and 0.0086 (ELOVL6); 0.0289 (FASN) and 0.0087 (FASN); 0.0377 (FABP5) and 0.0120 (FABP5); 0.1060 (HACD2) and 0.0087 (HACD2); 0.0192 (HADH) and 0.0112 (HADH); 0.0458 (HSL) and 0.0091 (HSL); 0.0258 (PLIN1) and 0.0111 (PLIN1); 0.0410 (PLIN4) and 0.0110 (PLIN4); 0.0310 (NDRG2) and 0.0091 (NDRG2); 0.0452 (TMEM120A) and 0.0129 (TMEM120A); 0.3753 (SERPINC1) and 0.0117 (SERPINC1). Differences with p values <0.05 were considered to be statistically significant. (DOC) [file pone.0246279.s003.doc]

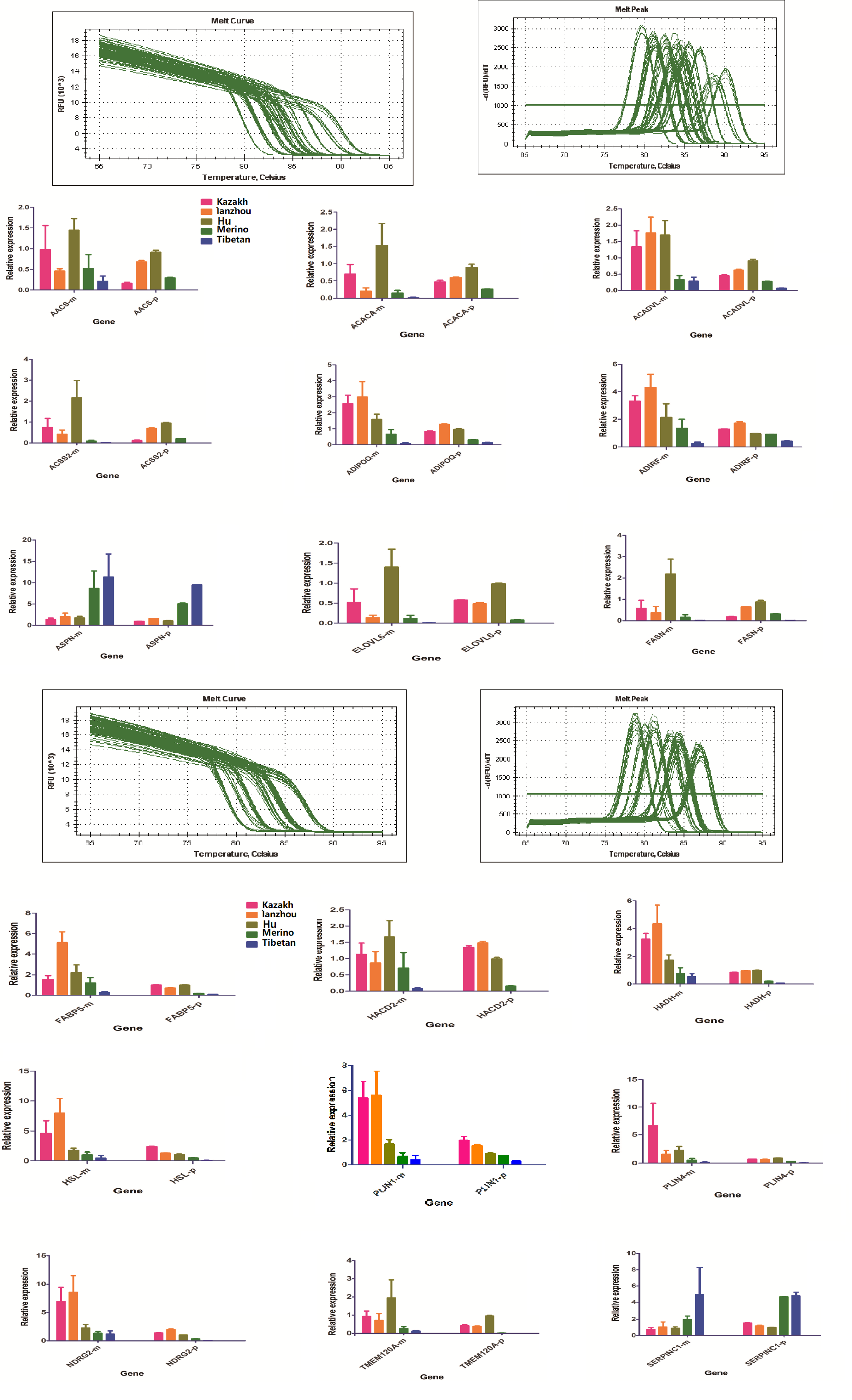


**S3 Fig. RT-qPCR verification quantitative proteomic of DEPs.** 18 proteins were selected, with GAPDH as the internal reference gene.

The *P* values of relative expression of gene and its protein: 0.1526(*AACS*) and 0.0087 (AACS); 0.0192 (*ACACA*) and 0.0086 (ACACA); 0.0337 (*ACADVL*) and 0.0091 (ACADVL); 0.0171 (*ACSS2*) and 0.0087 (ACSS2); 0.0197 (*ADIPOQ*) and 0.0101 (ADIPOQ); 0.0477 (*ADIRF*) and 0.0117 (ADIRF); 0.0337 (*ASPN*) and 0.0101 (ASPN) ; 0.0302 (*ELOVL6*) and 0.0086 (ELOVL6); 0.0289 (*FASN*) and 0.0087 (FASN); 0.0377 (*FABP5*) and 0.0120 (FABP5); 0.1060 (*HACD2*) and 0.0087 (HACD2); 0.0192 (*HADH*) and 0.0112 (HADH); 0.0458 (*HSL*) and 0.0091 (HSL); 0.0258 (*PLIN1*) and 0.0111 (PLIN1)；0.0410 (*PLIN4*) and 0.0110 (PLIN4); 0.0310 (*NDRG2*) and 0.0091 (NDRG2); 0.0452 (*TMEM120A*) and 0.0129 (TMEM120A); 0.3753 (*SERPINC1*) and 0.0117 (SERPINC1). Differences with *p* values <0.05 were considered to be statistically significant.
