# Supplementary material for: Quantitative proteomic analysis identified differentially expressed proteins with tail/rump fat deposition in Chinese thin- and fat-tailed lambs
Source: PLoS One. 2021 Feb 2;16(2):e0246279. doi: 10.1371/journal.pone.0246279 (PMC7853479; doi:10.1371/journal.pone.0246279)
Supplement: S1 Table — (DOC) [file pone.0246279.s004.doc]

**S1 Table.** The primer sequence for RT-qPCR

| Gene name | Reference Sequence | Primer sequences 5’-3’ | Products length (bp) |
| --- | --- | --- | --- |
| GAPDH-F | NM_001190390.1 | GATGAGATCAAGAAGGTGGT | 108 |
| GAPDH-R |  | GTGAGTGTCGCTGTTGAAGT |  |
| ACTB-f | NM_001009784.1 | CTGAGGCTCTCTTCCAGCCT | 110 |
| ACTB-r |  | GTAGAGGTCTTTGCGGATGT |  |
| PRKACA-F | NM_001009234.1 | TACATGGTCATGGAGTACGT | 117 |
| PRKACA-R |  | CTCAAAGGTCAGGACAATCT |  |
| FASN-F | XM_015098375.1 | GTCGGAGAACCTGGAGGAGT | 128 |
| FASN-R |  | ACAGGTCCTTCAGCTTGCCT |  |
| ACSS2-F | XM_004014514.3 | AACGTACTGGATCGAATTGT | 113 |
| ACSS2-R |  | ACCAGAAGCTCTTGGTATGT |  |
| HSD17β4-F | XM_004008685.2 | GCTGCTGATAAGGTTGTTGA | 102 |
| HSD17β4-R |  | CAGTGCTGTCTTCACAATCT |  |
| SERPINC1-F | NM_001009393.1 | TGCCTGTAACAACACACTCA | 116 |
| SERPINC1-R |  | GATAGAGTCGGCAATTCAGT |  |
| NDRG2-F | XM_015096901.1 | CAGCGATACTCACCTACCAT | 103 |
| NDRG2-R |  | CGCACGAAGTTCTGAATGAT |  |
| MAPK1-F | XM_012098101.1 | CAGTACTACGATCCAAGCGA | 124 |
| MAPK1-R |  | GCTGGAATCTAGCAGTCTCT |  |
| TMEM120A-F | XM_015104215.1 | CTTCGATATGGAGGCCTACT | 119 |
| TMEM120A-R |  | CGTACTCGTCTTTGTAGGCA |  |
| HSL-F | NM_001128154.1 | CTCGTGGCTCAACTCCTTCT | 102 |
| HSL-R |  | AGGGCTGCTTCAGACACACT |  |
| PLIN1-F | NM_001113773.1 | GACTCAGAGACCGAGGACAG | 125 |
| PLIN1-R |  | CACATCACGACTGAGACGGT |  |
| PLIN4-F | XM_015095882.1 | ATGTGAGCAGCCAACCAGAG | 103 |
| PLIN4-R |  | CTGTAGGCCACATGGAGCTG |  |
| HACD2-F | XM_015091732.1 | GTCTGTTAAACCATCTGCCT | 105 |
| HACD2-R |  | CGGCATATATTGTGAGCAGT |  |
| PNPLA2-F | NM_001308576.1 | GACAGTTCCACCAACATCCA | 138 |
| PNPLA2-R |  | GTAGCCCTGTTTGCACATCT |  |
| ACACA-F | XM_015098291.1 | CAGCACCGTCTGTGATGACT | 134 |
| ACACA-R |  | GCACAAACTCGATGACTCTG |  |
| FABP4-F | NM_001114667.1 | GAAGGTGCTCTGGTACAAGT | 113 |
| FABP4-R |  | GTAGCAGTGACACCGTTCAT |  |
| Gene name | Reference Sequence | Primer sequences 5’-3’ | Products length (bp) |
| FABP5-F | NM_001145180.1 | GCATTGGTTCAACATCAGGA | 114 |
| FABP5-R |  | GACCCGAGTACAGGTAACAT |  |
| AACS-F | NW_011942284.1 | GGCAGAATTCTGGAAATTCA | 123 |
| AACS-R |  | TCTGCGTAGTTGAGGCGACT |  |
| ACSL1-F | XM_015104563.1 | CCCTGGTGTATTTCTACGAT | 120 |
| ACSL1-R |  | GCCATTCATAGGGTTGGTCT |  |
| ADIRF-F | XM_012105457.2 | GCTCCTGGAAATCCTGTTCT | 115 |
| ADIRF-R |  | GGTAGTCTTGGCAACCTGGT |  |
| ADIPOQ-F | NM_001308565.1 | CCTGGTGAGAAGGGTGAGAA | 130 |
| ADIPOQ-R |  | CAGGTTCTCCCTTTCTGCCT |  |
| ACADVL-F | XM_004012636.2 | GCTGTGGACCAGTCTGATTC | 107 |
| ACADVL-R |  | GTGAGCTGGCCCTTGAACAT |  |
| HADH-F | XM_004009637.3 | ACATCCTGGCAAAATCTAGA | 107 |
| HADH-R |  | GGTCTTCGCCACAAACTCAT |  |
| ELOVL6-F | XM_012179509.2 | GAACTGAGGAAGCCTCTAGT | 132 |
| ELOVL6-R |  | CTGGTCACAAACTGAATGCT |  |
| ASPN-F | XM_004004077.3 | CCTCTGGATAACAATGGGAT | 121 |
| ASPN-R |  | GCTCCAGTAAAGTTGAAGGT |  |
