# Supplementary material for: Quantitative proteomic analysis identified differentially expressed proteins with tail/rump fat deposition in Chinese thin- and fat-tailed lambs
Source: PLoS One. 2021 Feb 2;16(2):e0246279. doi: 10.1371/journal.pone.0246279 (PMC7853479; doi:10.1371/journal.pone.0246279)
Supplement: S1 Raw image — (PDF) [file pone.0246279.s007.pdf]

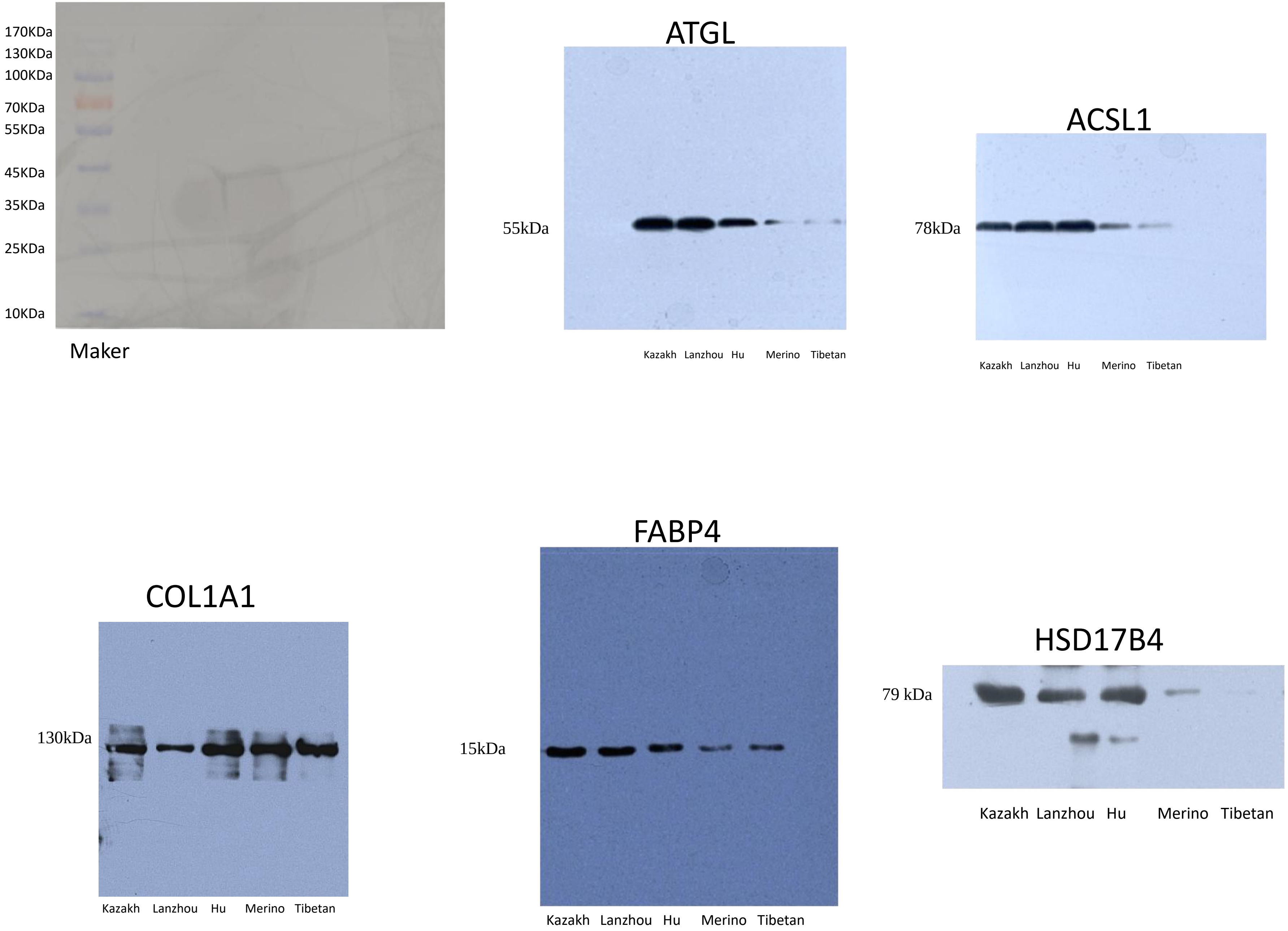

170KDa
130KDa
100KDa
70KDa
55KDa
45KDa
35KDa
25KDa
10KDa
Maker
ATGL
55kDa
Kazakh Lanzhou Hu Merino Tibetan
ACSL1
78kDa
Kazakh Lanzhou Hu Merino Tibetan
COL1A1
130kDa
Kazakh Lanzhou Hu Merino Tibetan
FABP4
15kDa
Kazakh Lanzhou Hu Merino Tibetan
HSD17B4
79 kDa
Kazakh Lanzhou Hu Merino Tibetan

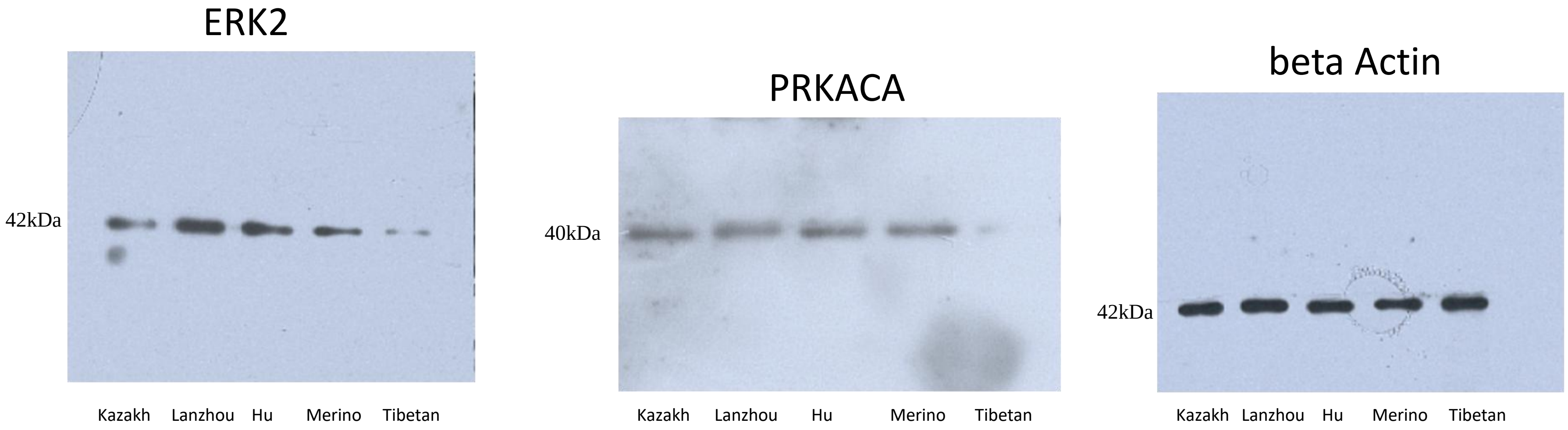
ERK2
42kDa
Kazakh
Lanzhou
Hu
Merino
Tibetan
PRKACA
40kDa
Kazakh
Lanzhou
Hu
Merino
Tibetan
beta Actin
42kDa
Kazakh
Lanzhou
Hu
Merino
Tibetan
