# Supplementary material for: Quantitative proteomic analysis identified differentially expressed proteins with tail/rump fat deposition in Chinese thin- and fat-tailed lambs
Source: PLoS One. 2021 Feb 2;16(2):e0246279. doi: 10.1371/journal.pone.0246279 (PMC7853479; doi:10.1371/journal.pone.0246279)
Supplement: Attachment — Submitted filename: Comments on Manuscript PONE-D-20-24379.docx [file pone.0246279.s008.docx]

Comments on manuscript PONE-D-20-24379 entitled (**Quantitative proteomic analysis identified differentially expressed proteins with tail/rump fat deposition in Chinese thin- and fat-tailed lambs**).

**Title:**

It is good and appropriate for the article.

**Abstract:**

Some information id required concerning the following:

1- Clearly indicate the aim of the study .
2- Indicate the breeds of sheep in this study.

**Introduction:**

The introduction is comprehensive and covers the topic of the study.

**Methodology:**

Some information id required concerning the following:

1- The components of the diet used in the current study and its chemical composition.
2- Lambs weights averages should be reported at the start and end of this study.
3- The period of study and the date of its work must be mentioned.
4- Some study parameters (adipose tissue staining, quantitative proteomic analysis and bioinformatics analysis ) should be measured based on scientific references.

**Results:**

The results are good and illustrated the figures in detail.

**Discussion:**

Unsatisfied in general. As compared with his results the authors shows the agreement or disagreement with literature without pay enough attention to discuss the reasons.

**Conclusions:**

It is good . It is drawn appropriately based on the data presented.

**References:**

1- Some references are very old.
2- Some references need to write total pages.
3- This reference (Zhou GX, Wang XL, Yuan C, Kang DJ, Xu XC, et al.) without Vol., No and total pages.
